# The Story of the Insane from Year to Year

**Published:** 1891-08-15

**Authors:** 


### THE STORY OF THE INSANE FROM YEAR TO YEAR.

(Fourth Series.)

[Annual Reports, Pamphlets, &c., should be addressed to The Editor, The Lodge, Porchester Square, London, W.]

### JOINT COUNTIES ASYLUM, CARMARTHEN.

The number of patients increased during the year from 529 to 543. The admissions were 78, the recoveries 18, and the deaths 42. The percentages of the recoveries and deaths calculated in the usual manner are respectively 24·6 and 7·92. Drink and heredity are known to have been the causes of the insanity in 28 cases, and were accurate information obtainable these causes would be seen to be more potent still. As usual the most of the deaths were from some form of brain disease; but phthisis accounts for five, and tuberculosis for two.

"The New Lunacy Act," says Dr. Hearder, "which came into force in May last, has largely added to the clerical duties of Asylum Superintendents, and the difficulty in construing various sections has led in this, and I have no doubt, in all asylumns, to much correspondence and considerable friction. It is not apparent that the additional work demanded by the Act will, or can in even the smallest degree, promote the welfare or safeguard the interests of the insane, while it must occupy time which might and should be more usefully employed." To most people outside of Parliament it must be an inscrutable mystery how the Act ever came to be framed, or how, having been framed, it ever passed the Houses.

The visitors say they have "much pleasure in again bearing testimony to the care and attention bestowed on the patients by Dr. Hearder, and to his able management of the establishment." The Commissioners in Lunacy say, "The duties of the Medical Superintendent have been largely increased by the Lunacy Act of 1890."

The balance in favour of the farm is £277. The weekly cost per head was a fraction under 8s.

### DERBY BOROUGH ASYLUM.

This asylum contained on the last day of the year 264 patients showing an increase of 78. The admissions were 156, the recoveries 36, and the deaths 36. Put into percentages the recoveries come out at the very satisfactory rate of 46 1, and the deaths at 14·6. The latter is higher than the average, and is accounted for by the high mortality from epilepsy and other brain diseases, and by the epidemic of influenza, which, in many cases, hastened death. Of the admissions 37 per cent. were due to drink and hereditary tendency.

Dr. Macphail's report is clear and concise. We notice that he has commenced a course of systematic instruction for attendants and nurses, and he is evidently determined to bring this newly-opened asylum up to a prominent place among kindred institutions. We are not surprised to find that the visitors conclude their report by saying, "The general management of the asylum under the superintendence of Dr. Macphail has been perfectly satisfactory." The farm accounts show a balance of £69 in favour of the farm.

The weekly charge to the Union has been reduced from 11s. 8d. to 10s. 6d., a sum which cannot be looked on as high if we remember the size of the asylum and its recent opening.

